# Supplementary material for: Retrospective Analysis of Prognostic Factors in Pediatric Patients with Adrenocortical Tumor from Unique Tertiary Center with Long-Term Follow-Up
Source: J Clin Med. 2022 Nov 9;11(22):6641. doi: 10.3390/jcm11226641 (PMC9692695; doi:10.3390/jcm11226641)
Supplement: Supplementary file 1 [file jcm-11-06641-s001.zip › jcm-1968919-supplementary.pdf]

## Supplementary Materials

**Supplementary File S1.** Materials and methods (genetic data)

In all patients, the Exon 10 of *TP53* (p.R337H) was firstly analyzed (due to be a hot spot region) by standard Sanger sequencing or enzymatic digestion (Hha I) from genomic DNA [12]. *TP53* regions, as the flanking intronic sequence of each exon, were amplified by polymerase chain reaction and sequenced on a 3730xl DNA Analyzer (Applied Biosystems). By the commercial multiplex ligation-dependent probe amplification (MLPA) kits (PO56 TP53 probemix MRC-Holland, Amsterdam, the Netherlands), large deletions of *TP53* were evaluated (as per the manufacturer's instructions) [18].

Total RNA was obtained from the frozen tumor fragments stored in liquid nitrogen using TRIzol (Invitrogen) reagent method. The agarose gel electrophoresis (1%) and the spectrophotometry evaluated the RNA integrity and concentration. The "High-capacity cDNA Archive" kit for RT (Applied Biosystems, Foster City, CA, USA) generated the cDNA. Using the TaqMan gene expression assays (Applied Biosystem), in the ABI PRISM 7000 Sequence Detector, the quantitative real-time PCR was performed. The assay IDs were BUB1B, Hs00177821_m1; PINK1, Hs00260868_m1; and DLGAP5, Hs007323_m1. A cycle threshold value in the linear range of amplification was selected for each sample in triplicate and normalized to human beta glucuronidase as endogenous control gene (4326320E). The differences of DCt (DCT DLGAP5–DCT PINK1) and (DCT BUB1B–DCT PINK1) were associated with pathological and clinical parameters [19].

The copy number of the *ATRX* was evaluated using the commercial assay "SALSA MLPA probemix P013-A2 ATRX" (MRC-Holland Amsterdam, Netherlands). It had 46 MLPA probes, with 37 probes for the *ATRX*, one for each exon and two probes for exons 9 and 35, and 9 reference probes for detecting different regions on the chromosome X. This probemix also contains 9 quality control fragments, 4 fragments for DNA quantity (Q-fragments), 2 fragments for denatured DNA (D-fragments), a reference fragment, a specific fragment for the X chromosome, and a specific fragment for the Y chromosome [14,20].

Subsequently, the copy number evaluation of the *ZNRF3* was performed by the commercial assay "SALSA MLPA probemix P476 *ZNRF3*" (MRC-Holland Amsterdam, Netherlands). It contains 42 MLPA probes, with 15 probes for the *ZNRF3*, 4 flanking probes for upstream genes and 5 flanking probes for downstream genes. It also had another 18 reference probes that target relatively stable copy number regions described in various types of neoplasms, such as the CAC [17]. It contains 9 quality control fragments, 4 fragments for DNA quantity, 2

fragments for denatured DNA, a reference fragment, a specific fragment for the X chromosome, and one specific fragment for the Y chromosome. The MLPA is interpreted by analysis of the dose-coefficient of the probes, which shows the results for autosomal or pseudo-autosomal chromosomes [14,17].

Regarding the *MMR*, the genomic DNA was analyzed using the research assay HNPCC MASTR Plus (Agilent Technologies, Santa Clara, CA) to identify single-nucleotide variants in *MLH1, MSH2, MSH6,* and *PMS2*. The MiSeq platform (Illumina, San Diego, CA) generated the sequences (in paired-end mode). Reads were aligned to the GRCh37/hg19 assembly of the human genome with the Burrows–Wheeler (BWA-mem) aligner. Variant calling (including single-nucleotide variants, small insertions, and deletions) was performed using the Freebayes platform. The resulting data (in variant call format) were annotated with ANNOVAR. The median coverage of the target bases was 7139.12X, with 100% of the targets having bases with ≥10× coverage. The variants were screened for rare variants (minor allele frequency < 0.1% in public and in-house databases) located in exonic regions and consensus splice site sequences. Afterward, variant filtration was performed to prioritize their pathogenic potential: loss-of-function variants and variants predicted to be pathogenic by multiple in silico programs. The sequencing reads carrying candidate variants were inspected visually using the Integrative Genomics Viewer (Broad Institute, Cambridge, MA). For classification of the variants, the databases gnomAD exome data, gnomAD genome data, and CllinVar, and the computational prediction sites DANN, DEOGEN2, EIGEN, FATHMM-MKL, M-CAP, MVP, MutationAssessor, MutationTaster, PrimateAI, REVEL, and SIFT were used. The Copy Number Targeted Resequencing Analysis performed the number variation analyses [21]. The guidelines of the American College of Medical Genetics and Genomics classified the variants according the Varsome platform [9,22].

*TP53*-R337H (rs121912664) and *XAF1*-E134* (rs146752602) variant frequencies were retrieved from the Genome Aggregation [12], Global Biobank Engine with genome-wide association analysis of 337,199 Caucasian British individuals [18], ABraOM ($n$ = 609) [18]; and SELA ($n$ = 739) databases; the two last databases contains exome sequences of cancer-free individuals from Southeastern Brazil. *TP53*-R337H and *XAF1*-E134* variant frequencies were also derived from the genomic information of the SJLIFE cohort comprising of 3006 adult survivors of childhood cancer [23]. *TP53* and *XAF1* variants were genotyped by PCR-restriction fragment-length polymorphism analysis and Sanger sequencing. The single-nucleotide polymorphisms (SNPs) and the microsatellite marker analyses mapped the *TP53* and *XAF1* loci [13].

**Supplementary File S2.** Prognostic factors with statistical significance, with their respective "p-values", hazard ratio and information coefficient

| Prognostic Factor | "p" value | Hazard Ratio | IC 95% |
|---|---|---|---|
| Age > 3 years | 0.03 | 2.5 | 1.07-5.8 |
| Higher serum levels of 11-deoxycortisol | < 0.001 | 2.9 | 0.9-1.2 |
| Tumor size ≥ 5 cm | < 0.05 | 5.3 | 1.03-5.4 |
| Tumor weight ≥ 200 g | < 0.001 | 16.2 | 0.9-2.1 |
| Weiss/Modified Weiss score ≥ 5 | < 0.05 | 6.2 | 1.2-8.3 |
| Wieneke index ≥ 3 | < 0.001 | 25.8 | 1.4-3.4 |
| MacFarlane index stages I-III | < 0.001 | 5.1 | 1.9-25.9 |
| Ki67 ≥ 15% | < 0.05 | 8.1 | 1.2-8.2 |
| (BUB1B-PINK1) < 6.95 | 0.062 | n.a. | n.a. |
| IGF-II receptor overexpression | 0.01 | 1.84 | n.a. |

IC: information coefficient; n.a.: not available;

**Supplementary File S3.** MacFarlane/ Sullivan modified

| Stage | Description |
|---|---|
| I | Tumor totally excised with negative margins. The tumor weight is < or equal than 200 g, there is no evidence of metastasis and the abnormal hormone levels return to normal after surgery. |
| II | Tumor totally excised with negative margins. The tumor weight is >200 g or there is persistence of abnormal hormone levels after surgery. Gross tumor excision with microscopic residual tumor. |
| III | Gross residual or inoperable tumor. |
| IV | Distant metastasis. |

**Supplementary File S4:** Genetic analysis about patients with pediatric adrenocortical tumor: the presence or the abscence of pathogenic variants of genes (*TP53, ZNRF3, ATRX, MLH1, MSH6* and *XAF1)*

| Patients # | Age (years ) | *TP53* Arg337His pathogenic variant | *ZNRF3* somatic pathogenic variant | *ATRX* somatic pathogenic variant | *MLH1* germline pathogenic/ likely pathogenic variant | *MSH6* germline pathogenic variant | *XAF1 E134* pathogenic variant |
|---|---|---|---|---|---|---|---|
| 1 | 1.4 | Yes | n.a. | n.a. | No | No | n.a. |
| 2 | 2.6 | Yes | n.a. | n.a. | No | No | Yes |
| 3 | 0.1 | Yes | no | No | No | No | Yes |
| 4 | 1 | No | n.a. | n.a. | No | No | Yes |
| 5 | 2.1 | Yes | n.a. | n.a. | No | No | No |
| 6 | 3.7 | Yes | n.a. | n.a. | n.a. | n.a. | n.a. |
| 7 | 2.3 | No | no | No | No | No | Yes |
| 8 | 2.2 | Yes | no | No | No | No | Yes |
| 9 | 2.6 | n.a. | n.a. | n.a. | No | No | n.a. |
| 10 | 2.1 | Yes | n.a. | n.a. | Yes | No | Yes |
| 11 | 2.2 | No | no | No | Yes | No | Yes |
| 12 | 2.3 | n.a. | n.a. | n.a. | No | No | n.a. |
| 13 | 1.1 | n.a. | n.a. | n.a. | n.a. | n.a. | n.a. |
| 14 | 0.8 | Yes | n.a. | n.a. | n.a. | n.a. | n.a. |
| 15 | 1 | Yes | n.a. | n.a. | n.a. | n.a. | n.a. |
| 16 | 3.4 | Yes | n.a. | n.a. | n.a. | n.a. | n.a. |

| | | | | | | | |
|---|---|---|---|---|---|---|---|
| 17 | 0.8 | Yes | yes | Yes | No | No | Yes |
| 18 | 1.4 | Yes | n.a. | n.a. | No | No | n.a. |
| 19 | 1 | Yes | n.a. | n.a. | No | No | Yes |
| 20 | 1 | n.a. | n.a. | n.a. | n.a. | n.a. | n.a. |
| 21 | n.a. | n.a. | n.a. | n.a. | No | No | n.a. |
| 22 | 3 | n.a. | n.a. | n.a. | n.a. | n.a. | n.a. |
| 23 | 0.1 | Yes | n.a. | n.a. | n.a. | n.a. | n.a. |
| 24 | 3.3 | No | n.a. | n.a. | n.a. | n.a. | n.a. |
| 25 | 3.3 | Yes | no | Yes | No | VUS | Yes |
| 26 | 1 | Yes | no | Yes | No | No | Yes |
| 27 | 2.6 | n.a. | n.a. | n.a. | n.a. | n.a. | n.a. |
| 28 | 1.9 | n.a. | n.a. | n.a. | n.a. | n.a. | n.a. |
| 29 | 2.6 | No | n.a. | n.a. | n.a. | n.a. | n.a. |
| 30 | 1.9 | n.a. | n.a. | n.a. | No | No | n.a. |
| 31 | 2.6 | n.a. | n.a. | n.a. | n.a. | n.a. | n.a. |
| 32 | 1.3 | Yes | n.a. | n.a. | n.a. | n.a. | n.a. |
| 33 | 2 | Yes | n.a. | n.a. | No | No | Yes |
| 34 | 0.11 | Yes | n.a. | n.a. | No | No | n.a. |
| 35 | 2.1 | No | n.a. | n.a. | n.a. | n.a. | n.a. |
| 36 | 3.1 | Yes | yes | No | No | No | n.a. |
| 37 | 0.11 | Yes | n.a. | n.a. | No | No | Yes |
| 38 | 3.6 | Yes | n.a. | n.a. | No | No | Yes |
| 39 | 1.3 | Yes | n.a. | n.a. | No | No | Yes |
| 40 | 2.8 | Yes | no | No | No | No | n.a. |
| 41 | 1.6 | Yes | n.a. | n.a. | No | No | n.a. |
| 42 | 2.9 | n.a. | n.a. | n.a. | n.a. | n.a. | n.a. |
| 43 | 1.9 | n.a. | n.a. | n.a. | n.a. | n.a. | n.a. |
| 44 | 3 | n.a. | n.a. | n.a. | n.a. | n.a. | n.a. |
| 45 | 1.1 | No | n.a. | n.a. | n.a. | n.a. | n.a. |
| 46 | 1.8 | Yes | n.a. | n.a. | n.a. | n.a. | n.a. |
| 47 | 1 | No | n.a. | n.a. | n.a. | n.a. | n.a. |
| 48 | 0.7 | n.a. | n.a. | n.a. | n.a. | n.a. | n.a. |
| 49 | 1.11 | No | n.a. | n.a. | n.a. | n.a. | n.a. |
| 50 | 3 | n.a. | n.a. | n.a. | No | No | n.a. |
| 51 | 3.11 | n.a. | n.a. | n.a. | n.a. | n.a. | n.a. |
| 52 | 1 | n.a. | n.a. | n.a. | n.a. | n.a. | n.a. |
| 53 | 2 | Yes | n.a. | n.a. | n.a. | n.a. | n.a. |
| 54 | 1 | Yes | n.a. | n.a. | No | No | n.a. |
| 55 | 1.7 | No | n.a. | n.a. | n.a. | n.a. | n.a. |
| 56 | 3 | Yes | n.a. | n.a. | No | No | Yes |
| 57 | 2.7 | Yes | n.a. | n.a. | No | No | No |
| 58 | 2.1 | Yes | n.a. | n.a. | No | No | n.a. |
| 59 | 2.3 | Yes | n.a. | n.a. | No | No | Yes |
| 60 | 0.11 | No | n.a. | n.a. | n.a. | n.a. | n.a. |
| 61 | 3 | No | n.a. | n.a. | No | No | n.a. |
| 62 | 3 | n.a. | n.a. | n.a. | n.a. | n.a. | n.a. |
| 63 | 1.9 | Yes | n.a. | n.a. | No | No | Yes |
| 64 | 2.6 | n.a. | No | No | No | No | n.a. |
| 65 | 0.9 | n.a. | n.a. | n.a. | No | No | n.a. |
| 66 | 2 | n.a. | n.a. | n.a. | n.a. | n.a. | n.a. |

| | | | | | | | |
|---|---|---|---|---|---|---|---|
| 67 | 2 | No | n.a. | n.a. | n.a. | n.a. | n.a. |
| 68 | 7 | No | n.a. | n.a. | n.a. | n.a. | n.a. |
| 69 | 5 | n.a. | n.a. | n.a. | n.a. | n.a. | n.a. |
| 70 | 4.6 | No | n.a. | n.a. | n.a. | n.a. | n.a. |
| 71 | 7 | no | n.a. | n.a. | n.a. | n.a. | n.a. |
| 72 | 3.3 | n.a. | Yes | No | n.a. | n.a. | n.a. |
| 73 | 5 | n.a. | n.a. | n.a. | n.a. | n.a. | n.a. |
| 74 | 4 | n.a. | n.a. | n.a. | No | No | n.a. |
| 75 | 9.6 | No | n.a. | n.a. | n.a. | n.a. | n.a. |
| 76 | 6.2 | No | n.a. | n.a. | n.a. | n.a. | n.a. |
| 77 | 10 | No | n.a. | n.a. | n.a. | n.a. | n.a. |
| 78 | 6 | No | n.a. | n.a. | n.a. | n.a. | n.a. |
| 79 | 6 | Yes | n.a. | n.a. | No | No | Yes |
| 80 | 6 | Yes | Yes | No | No | No | Yes |
| 81 | 11.5 | Yes | n.a. | n.a. | No | Yes | n.a. |
| 82 | 9.6 | no | n.a. | n.a. | n.a. | n.a. | n.a. |
| 83 | 16.1 | n.a. | n.a. | n.a. | n.a. | n.a. | n.a. |
| 84 | 15 | n.a. | n.a. | n.a. | n.a. | n.a. | n.a. |
| 85 | 14.5 | n.a. | n.a. | n.a. | n.a. | n.a. | n.a. |
| 86 | 17 | n.a. | n.a. | n.a. | n.a. | n.a. | n.a. |
| 87 | 13 | yes | No | No | No | No | Yes |
| 88 | 16 | n.a. | n.a. | n.a. | n.a. | n.a. | n.a. |
| 89 | 14 | n.a. | n.a. | n.a. | n.a. | n.a. | n.a. |
| 90 | 18 | no | n.a. | n.a. | No | No | No |
| 91 | 17.4 | n.a. | n.a. | n.a. | n.a. | n.a. | n.a. |
| 92 | 17 | n.a. | n.a. | n.a. | n.a. | n.a. | n.a. |
| 93 | 17 | n.a. | n.a. | n.a. | n.a. | n.a. | n.a. |
| 94 | 18 | n.a. | n.a. | n.a. | n.a. | n.a. | n.a. |
| 95 | 15.7 | n.a. | n.a. | n.a. | n.a. | n.a. | n.a. |

n.a.: not available; yes: presence of pathogenic variant of the gene; no: abscence of pathogenic variant of the gene; VUS: Variant of uncertain significance.
